# FACTORS ASSOCIATED WITH THE USE OF A PERSONALIZED MUSIC INTERVENTION FOR NURSING HOME RESIDENTS WITH DEMENTIA

**DOI:** 10.1093/geroni/igad104.2133

**Published:** 2023-12-21

**Authors:** Ryan Conard, Ellen McCreedy, Laura Dionne, Vincent Mor

**Affiliations:** Brown University, Providence, Rhode Island, United States; Brown University School of Public Health, Providence, Rhode Island, United States; Brown University School of Public Health, Providence, Rhode Island, United States; Brown University School of Public Health, Providence, Rhode Island, United States

## Abstract

Music offers a promising non-pharmacological alternative for managing behavioral dysregulations in people with Alzheimer’s disease and other dementias (ADRD). Using data from an embedded, pragmatic trial (ePCT) of a personalized music intervention for nursing home (NH) residents with ADRD, we examined resident and NH characteristics associated with exposure to the intervention and dose of music received. Participants were enrolled from 54 NHs (27 treatment, 27 control) between June 2019 and February 2020. The intervention was resident-preferred music delivered at early signs of agitation. Intervention dose was calculated by multiplying song duration and number of plays, averaged over days exposed. Facility and resident-level characteristics were identified using the Minimum Data Set and the Certification and Survey Provider Enhanced Reports. A mixed-effects hurdle model was used. 483 residents participated (67.7% female, mean age 79.8±12.2 years). Female residents (p=0.04) taking antipsychotic medications (p=0.06) were more likely to receive the intervention, as were residents from NHs with greater nursing involvement (p=0.02). Residents with greater health instability received a greater dose (p=0.04). In this ePCT of a personalized music intervention, NHs with more nursing engagement had greater use of the intervention and appropriately chose residents with antipsychotic use to participate. After adjusting for initial selection, staff used the intervention more frequently with residents who had a higher likelihood of death in the next six months, potentially indicating the beneficial use for comfort at the end of life. Our findings offer insights into future tailoring of personalized music interventions to increase the likelihood of successful implementation.

